# Antimicrobial Resistance in Bovine Respiratory Disease Pathogens: A Systematic Review and Analysis of the Published Literature

**DOI:** 10.3390/ani15121789

**Published:** 2025-06-18

**Authors:** Brian V. Lubbers, Andi Warren, Bradley J. White, Siddartha Torres, Pedro Rodriguez

**Affiliations:** 1 Department of Clinical Sciences and Beef Cattle Institute, Kansas State University, Manhattan, KS 66506, USA; bwhite@vet.k-state.edu; 2 Veterinary Medical Library, Kansas State University, Manhattan, KS 66506, USA; aparrish@vet.k-state.edu; 3 Merck Animal Health, Rahway, NJ 07065, USA; siddartha.torres@merck.com; 4 Merck Animal Health, Kikland, QC H9H 4M7, Canada; pedro.rodriguez.fernandez@merck.com

**Keywords:** antimicrobial resistance, antimicrobial susceptibility testing, bovine respiratory disease, florfenicol, tildipirosin, tulathromycin

## Abstract

Bovine respiratory disease (BRD) is the most significant disease challenge in the feeder cattle industry in North America. Antimicrobials are commonly administered in BRD cases due to the role of bacterial pathogens. However, reports of antimicrobial resistance (AMR) in these pathogens raise concerns regarding their long-term effectiveness to treat BRD cases. A systematic literature review was conducted to summarize AMR in North American BRD pathogens and determine if changes in AMR prevalence over time could be identified for antimicrobials routinely used for treatment and control of BRD. Generalized linear models were used to test associations between the proportion of resistant isolates for each of the three bacterial agents and antimicrobial agent, isolation year, and timing of sample collection. The antimicrobial agent and timing of sample collection were significantly associated with the proportion of antimicrobial resistant isolates, with increased probability of resistance to tulathromycin seen in *Mannheimia haemolytica* (24.08%) and *Histophilus somni* (8.19%) and increased resistance to tildipirosin in *Pasteurella multocida* (21.48%), while samples collected at arrival demonstrated a lower proportions of resistant bacteria. Trends over time could not be evaluated due to the limited number of published studies. These findings highlight the differences in AMR seen between antimicrobials, BRD pathogens, and sample types and emphasize the need for continued AMR surveillance.

## Simple Summary

Bovine respiratory disease, or cattle pneumonia, is the most common disease affecting cattle in North American feedlots. Antibiotics are commonly used to treat the underlying bacterial infections that cause cattle pneumonia; however, antimicrobial resistance in these bacteria may threaten the usefulness of antibiotics labeled to treat this disease. A review of scientific articles published between 2015 and 2024 was conducted to determine (1) if changes in antimicrobial resistance over time have occurred and (2) if antimicrobial resistance is common in the bacteria causing cattle pneumonia. Trends over time could not be evaluated due to the limited number of published studies. This review and analysis does suggest that antimicrobial resistance for three approved antibiotics varies considerably among the most common bacteria that cause cattle pneumonia and timing of sample collection is associated with the prevalence of resistance. These results provide veterinarians with valuable information regarding the current state of antimicrobial resistance in bacteria that cause cattle pneumonia and enable them to make science-based antimicrobial stewardship decisions in cases of cattle pneumonia.

## 1. Introduction

Bovine respiratory disease (BRD) is a clinical syndrome describing the end result of a complex cascade of host, environment, and pathogen interactions. Typically, increases in BRD incidence are seen shortly after management changes such as weaning, transport, or commingling (or all of these combined). Our current understanding of the BRD process is that these events lead to a weakening of the host immune system that results in opportunistic infection by bacteria residing in the bovine nasopharynx, such as *Mannheimia haemolytica*, *Pasteurella multocida,* and/or *Histophilus somni* [1]. Due to the importance of these bacterial pathogens in BRD pathogenesis, antimicrobials continue to be the mainstay of therapy [2].

As antimicrobials play a critical role in the control and treatment of BRD, antimicrobial resistance (AMR) is a serious threat to both animal health and productivity [3]. Antimicrobial resistance in the Pasteurellaceae family is not a new phenomenon, as published reports describing AMR in BRD pathogens can be found dating back to the 1970s [4]. However, reports of AMR in BRD pathogens appear to be increasing in published literature. A 2015 review evaluating AMR in BRD pathogens conducted by DeDonder and Apley concluded that a quantitative assessment was not possible, but that “following these reports chronologically shows an apparent trend of a decrease in susceptibility of the 3 major BRD pathogens…” [5].

Therefore, the primary objective of this literature review and analysis was to determine if antimicrobial resistance in BRD pathogens is changing over time. The secondary objective was to summarize the current state of AMR for North American isolates of *Mannheimia haemolytica*, *Pasteurella multocida,* and *Histophilus somni* to florfenicol, tildipirosin, and tulathromycin. The results of this literature review highlight the important differences that exist in the prevalence of AMR between primary BRD pathogens and commonly used antimicrobials.

## 2. Materials and Methods

A comprehensive literature search was conducted using PubMed to identify studies relevant to the objectives of this systematic review. The search focused on publications from 2015 to 2024 to ensure the inclusion of contemporary research. A detailed search strategy was developed iteratively, combining keywords and Medical Subject Headings (MeSH) terms using Boolean operators (AND, OR) to create a broad yet targeted query. Key terms (and variants of these terms) included “cattle”, “cow”, “anti-microbial resistance”, “mannheimia haemolytica”, “pasteurella multocida”, and specific drugs such as “Tulathromycin”, “Tildipirosin”, and “Florfenicol”. The queries were designed to capture articles addressing bacterial resistance and related interventions in beef cattle while excluding studies outside the scope of the review. The search process was refined through multiple iterations to balance sensitivity and specificity, resulting in a comprehensive dataset. To enhance specificity, certain terms were deliberately excluded after the initial iterations. Excluded terms included “dairy cattle”, “dairy”, “fungal resistance”, and their variations, as these were not relevant to the study’s focus on bacterial resistance in beef cattle. Filters were applied to limit the results to studies published within the specified date range of 2015 to 2024. Additionally, all duplicate publications were identified and removed to ensure that only unique records were included in the screening process.

Exact search methodology, including terms used in each iteration of the search and the resulting number of articles retrieved are detailed in Supplementary Table S1. A supplemental search of the grey literature (Google Scholar) was conducted using the search string “antimicrobial resistance susceptibility mannheimia pasteurella histophilus cattle”.

A title and abstract review was conducted by a single researcher [BVL] in Jan 2025 to identify studies that were relevant to the study objectives. Articles printed in languages other than English and articles presenting AMR data for isolates collected from species other than cattle or outside of North America (United States, Canada and Mexico) were excluded. A complete manuscript review was subsequently completed. Articles that did not use standardized phenotypic antimicrobial susceptibility testing (AST) methods, including use of current clinical breakpoints for *M. haemolytica* and *P. multocida* (presentation of raw MIC data, which could be converted to % resistance, did not warrant exclusion), and articles that presented only summary AST measures (MIC50/MIC90) were excluded from the final analysis. Articles for which AST had been reported as part of a previous publication and those which summarized AST data over extended time periods (>5 years) were also excluded as they did not align with specific objectives of this literature review. This systematic and reproducible approach adhered to established guidelines for conducting systematic reviews in veterinary medicine.

For published manuscripts that met the above criteria, the following data were extracted and formatted in spreadsheets for subsequent analysis:

- Author(s);
- Article title;
- Journal;
- Year of publication;
- Timing of sample collection;
- Bacterial genus species;
- Year(s) during which isolates were collected;
- Number of bacterial isolates tested;
- Number of bacterial isolates defined as “resistant” to
  ○ Florfenicol
  ○ Tildipirosin
  ○ Tulathromycin

In most published manuscripts, isolate collection occurred over multiple years. For analysis purposes, study year was defined as the year when isolate collection ended. When the period of isolate collection was not specified in the manuscript, the year of manuscript publication minus 2 years was used as a surrogate timepoint.

Published manuscripts that met all inclusion criteria for this systematic review largely fell into two categories—cross-sectional studies (diagnostic laboratory data summaries/prospective AMR surveillance) and longitudinal studies evaluating the effect of antimicrobial exposure on prevalence of AMR in a defined animal population; therefore, the authors considered traditional sources of bias in systematic reviews (“intention to treat” and “per-protocol” effects) to be non-applicable or have minimal impacts on the results of this literature review [6]. However, antimicrobial exposure (or lack of) was deemed to have considerable potential to bias the prevalence of AMR in a population of bacterial isolates. In general, the included studies did not contain complete details on antimicrobial exposure at the individual isolate level and the current review uses sample timing as a proxy for the likelihood of antimicrobial exposure. This review used the following sample timing categories as described by the publishing authors: samples collected at arrival to the study facility [lowest antimicrobial selection pressure], samples from diagnostic submissions (primarily, but not exclusively necropsy) [high likelihood of antimicrobial exposure], samples collected (by study design) at a timepoint after arrival (typically 2 weeks) at the study facility [typically a single known antimicrobial exposure], and samples that consisted of a combination of arrival and post-arrival samples [unknown antimicrobial exposure].

Generalized linear models (GLMER package in R [version 4.3.2]) were used to model potential associations between the proportion of resistant isolates for each bacterial species and antimicrobial agent, isolation year, and timing of sample collection. All key variables were retained in each model as they were portions of the experimental design and reporting. Interactions among all variables were tested and publication was included as a random effect in the analysis to account for lack of independence in samples reported in the same study. Estimated model means are only reported for factors deemed significant (*p* < 0.05) in the final model. Interactions were not included in the final model due to sparse data and lack of model convergence.

## 3. Results

**Figure 1:**
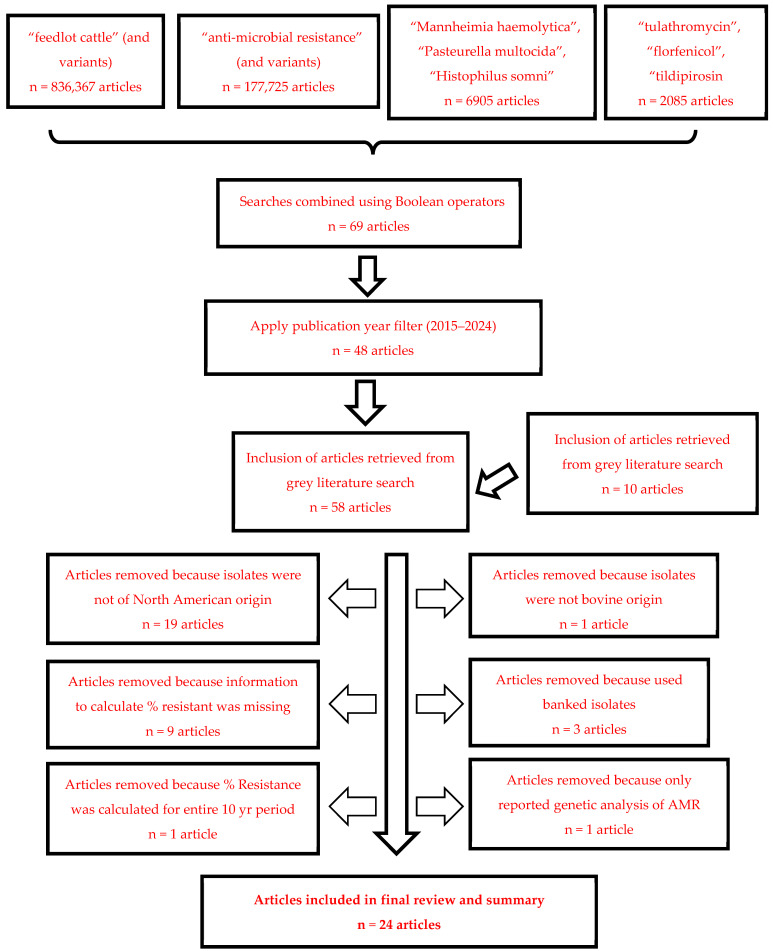
Literature search results following step-wise application of exclusion criteria.

The search itself was conducted systematically in multiple stages. An initial search for “feedlot cattle” and all variations in the term yielded 836,367 results. A subsequent search for “anti-microbial resistance”, and similar terms produced 177,725 articles. This was followed by a search for bacterial species of interest, which resulted in 6905 articles, and finally, a search for the specific drugs, yielding 2085 articles. These searches were then combined, using Boolean operators to integrate the cattle, bacteria, anti-microbial resistance, and drug parameters into a single query. This step refined the results to 69 articles. Adding a publication year filter (2015–2024) further narrowed the results to 48 articles. An additional 10 articles were retrieved from the “grey” literature for a total of 58 articles relevant to assessing temporal changes in AMR in BRD pathogens. Search strategy results are depicted in Figure 1.

Twenty manuscripts were excluded from further consideration following title/abstract screening for the following reasons:

- Abstract indicated that the isolates were not bovine origin (1 manuscript excluded)
- Abstract indicated that the isolates did not originate from North America (19 manuscripts excluded)

A complete manuscript review was conducted on the remaining 38 articles to evaluate suitability for inclusion in this literature review. Given the objectives of this review, 14 additional manuscripts were excluded for the following reasons:

- The manuscript did not contain any AST data or the AST data was presented as a summary measure (MIC_50_/MIC_90_) that prohibited the calculation of % resistant (nine manuscripts excluded);
- The manuscript used banked isolates and did not indicate when the isolates were recovered or if AST data for the isolates had been previously reported in other studies (three manuscripts removed);
- The manuscript conducted only genetic analysis of AMR genes in BRD pathogens without reporting phenotypic AST data (one manuscript excluded);
- The manuscript reported a single estimate of the percent of isolates resistant for a 10 yr period (one manuscript excluded).

Twenty-four manuscripts met the desired criteria for inclusion and provided data relevant to the overall objective of this literature review. Nine of these studies reported isolates collected during a single calendar year. Nine additional studies reported isolates that were collected during two consecutive years, although that could indicate collection starting in the fall months (e.g., Sept, Oct, Nov) that carried into the early months of the next year (e.g., Jan, Feb, Mar). Four studies either collected or summarized data over a period of three or more years. Two studies did not indicate the year of isolate collection and the year of publication minus two years was used as a surrogate timepoint for this analysis.

**Figure 2:**
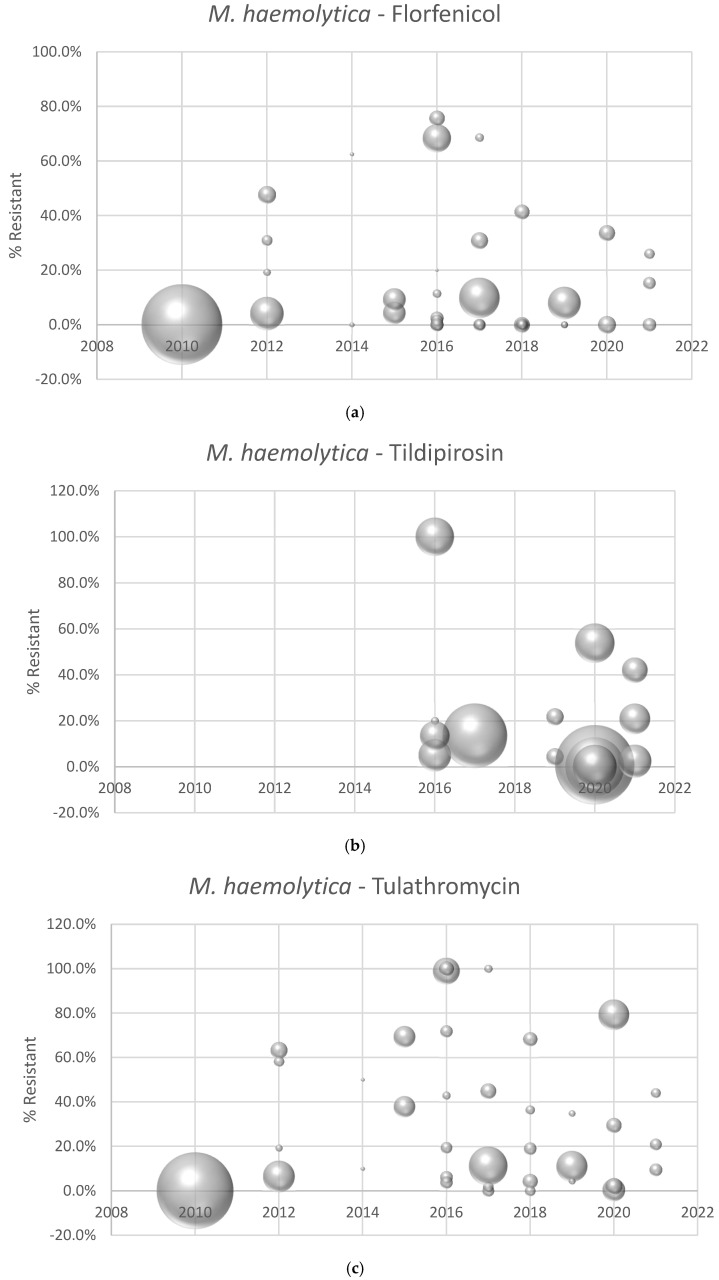
(**a**) Prevalence of **florfenicol** resistance reported by individual manuscript and year of isolate collection. Each point represents the percent of resistant isolates estimated from an individual manuscript. Bubble sizes represent the relative number of isolates in each published study. (**b**) Prevalence of **tildipirosin** resistance reported by individual manuscript and year of isolate collection. Each point represents the percent of resistant isolates estimated from an individual manuscript. Bubble sizes represent the relative number of isolates in each published study. (**c**) Prevalence of **tulathromycin** resistance reported by individual manuscript and year of isolate collection. Each point represents the percent of resistant isolates estimated from an individual manuscript. Bubble sizes represent the relative number of isolates in each published study.

Antimicrobial susceptibility data were not reported for all three BRD pathogens (*M. haemolytica*, *P. multocida*, and *H. somni*) for all three antimicrobials (tulathromycin, florfenicol and tildipirosin) in each manuscript. Table 1a–c outlines the number of manuscripts reporting AST data for the various pathogen-antimicrobial combinations and the minimum, maximum, and median number of isolates tested. The prevalence of resistance reported in individual manuscripts for *M. haemolytica* to florfenicol, tildipirosin, and tulathromycin is depicted in Figure 2a–c. The prevalence of resistance by individual manuscript for *P. multocida* and *H. somni* can be found in Supplementary Figures S1a–c and S2a–c.

There was significant heterogeneity in the study populations and methodologies used in the 24 published manuscripts. Most of these studies evaluated AMR in beef cattle, but two studies used isolates recovered from dairy calves and two studies were diagnostic laboratory summaries that likely contained isolates recovered from both beef and dairy animals. Eleven publications used a longitudinal study design that collected isolates during the feeding phase, typically at arrival in the feeding facility (prior to metaphylactic administration of antimicrobials) and at some subsequent timepoint, although the follow-up sample ranged from a few days up to 120 days in the various studies.

In the statistical analysis, separate models were tested for the three bacterial pathogens, and all models revealed significant associations between drug and sample collection timing with the probability of resistance (Table 2). Year was not included in the final analysis due to lack of model convergence. In *M. haemolytica* isolates, higher (*p* < 0.05) resistance was noted to tulathromycin (24.1% ± 0.06; model adjusted probability ± SE) compared to both florfenicol and tildipirosin (7.0% ± 0.02, 6.3% ± 0.02, respectively), but no differences were noted between florfenicol and tildipirosin. Sampling at the post-arrival timepoint resulted in higher probability of resistance (33.2% ± 0.09) compared to arrival (2.6% ± 0.01) and arrival and post-arrival (0.08% ± 0.001) but was not different than diagnostic submissions (18.9% ± 0.16). In *P. multocida* isolates, higher (*p* < 0.05) resistance was noted to tildipirosin (21.5% ± 0.07) compared to tulathromycin (10.5% ± 0.03), which was also higher than florfenicol resistance (4.8% ± 0.02). Sampling at the post-arrival timepoint illustrated higher (*p* < 0.05) resistance (17.9% ± 0.07) compared to arrival (2.9% ± 0.01) and arrival and post-arrival (0.03% ± 0.004) but was not different than diagnostic submissions (17.8% ± 0.07). In *H. somni* isolates, higher (*p* < 0.05) resistance was noted to tulathromycin (8.2% ± 0.03; model adjusted probability ± SE) compared to tildipirosin (5.1% ± 0.02), which was also higher than florfenicol resistance (1.2% ± 0.01). Sampling at the post-arrival timepoint illustrated higher (*p* < 0.05) resistance (4.2% ± 0.02) compared to arrival (1.2% ± 0.006), but arrival and post-arrival (1.7% ± 0.02) and diagnostic submissions (9.0% ± 0.08) were not different from each other or any other sample collection timepoint.

## 4. Discussion

Considering the scope of this literature review (10 years), there were a limited number of published manuscripts that provide insight on AMR in North American BRD pathogens for florfenicol, tildipirosin, and tulathromycin. For any specific antimicrobial–pathogen combination, there were less than 25 published manuscripts that met the inclusion criteria for this review. Given the significance of BRD in cattle health and production and the critical importance of antimicrobials in treating BRD, this is an incredibly limited dataset for informing treatment decisions and antimicrobial stewardship policy. Expanding this literature review to earlier publication years would have inevitably led to additional manuscripts; however, antimicrobial resistance is an evolving situation, and prudent decision-making requires contemporaneous information.

One of the primary objectives of this literature review was to evaluate if discernible trends in AMR for the three primary bacterial BRD pathogens could be detected by analysis of published literature. Year of bacterial isolation could not be included in the final analysis. This is not surprising, given the paucity of published reports, and it is likely that any review of the available scientific literature would be underpowered to detect even meaningful trends in AMR among the BRD pathogens during the specified review period. This literature summary does indicate that the overall prevalence of AMR in BRD pathogens is low over this time period; however, AMR is indeed present in BRD pathogens. For example, the prevalence of AMR is relatively high (>50% of isolates resistant) in nearly 30% of the individual study populations for *M. haemolytica*—tulathromycin. The variability in AMR seen between studies may be related to timing of sample collection, as the current analysis indicates that timing of sample collection is significantly associated with the prevalence of AMR in BRD pathogens.

For all three bacterial pathogens, isolates from samples collected post-arrival had a statistically higher prevalence of AMR compared to isolates collected at arrival. One potential explanation for this association is that timing of sample collection is serving as a proxy for antimicrobial exposure. A previously published report demonstrated that the percentage of resistant isolates recovered post-mortem increased with the number of antimicrobial treatments that an animal received [31]. In many of the reviewed studies, arrival samples were collected prior to the administration of an antimicrobial for control of BRD, while the post-arrival samples and diagnostic submissions were collected after this exposure occurred. In light of the AMR variability noted between pathogens in this review, this antimicrobial exposure does not appear to have the same effect on all pathogens and should be considered in future BRD AMR research studies.

This literature review demonstrates that these three bacterial pathogens do not show the same propensity for acquiring and/or expressing AMR phenotypes. This finding is not unique as previously published summaries of AMR in BRD pathogens have reported a higher prevalence of resistance in *M. haemolytica* relative to AMR seen in *P. multocida* and *H. somni* [32]. The scientific community needs to be more granular in discussing resistance in “BRD pathogens”, as this review reiterates that AMR patterns in *M. haemolytica* are not reflective of AMR in *P. multocida* or *H. somni*. This is also true when describing AMR to particular antimicrobials. These differences between pathogens and antimicrobials in the proportion of resistant isolates warrants further investigation. These results cannot wholly be explained by simple exposure and selection pressure, as these three bacterial pathogens would have similar exposure to antimicrobials, i.e., veterinarians are treating “BRD”, not “*M. haemolytica*”. While the acquisition of resistance to multiple antimicrobials, such as occurs with integrative-conjugative elements, may partially explain these differences, other antimicrobial–pathogen–host factors may play an important role in the selection for and dissemination of AMR throughout the North American cattle industry [33].

There are several limitations to this literature review that deserve further consideration. First of all, the data extracted from the individual published reports are not part of an integrated surveillance study, they are truly disparate datapoints. These studies were not designed to contribute to an analysis over time and represent a very heterogenous mix of animal populations, geographic locations, antimicrobial exposures and number of isolates. For example, 11 studies used a longitudinal study design that collected isolates from a population of cattle at multiple timepoints; however, the collection timepoints varied considerably. Most studies collected the first respiratory sample at feedlot arrival, but one study collected this sample at the farm of origin, approximately 6 months before calves were transported to the feedlot [17]. Many of these studies collected the second sample between 7 and 14 days after animals arrived in the feedlot (and were exposed to metaphylactic administration of antimicrobials). However, one study collected the follow-up samples at any time during the feeding period that an animal was diagnosed with BRD (some animals were sampled > 180 days on feed in this study) [26]. Clearly, antimicrobial exposure has the potential to impact the estimate of resistance; however, the lapse of time between exposure and microbiological sampling could impact this estimate as well.

The lack of integration between studies also results in an unequal number of reports over time, thus limiting conclusions that can be made regarding temporal trends. In 2 of the 24 studies summarized here, the time period of bacterial isolation was not specified which introduces uncertainty to the analysis and may have further limited our ability to evaluate temporal patterns of AMR in BRD pathogens. Another limitation to this review is that individual studies were not excluded based on laboratory methodologies. Phenotypic antimicrobial susceptibility test results can be impacted by alterations to standardized methods, particularly for macrolide class antimicrobials (tildipirosin and tulathromycin) [34]. While many of the articles included in this review referenced Clinical and Laboratory Standards Institute methodologies, sufficient testing details were not provided in the individual publications to enable evaluation and/or exclusion.

## 5. Conclusions

Overall, a clear understanding of AMR (both the current state, as well as trends over time) in *M. haemolytica*, *P. multocida,* and *H. somni* enables veterinarians to make informed decisions regarding the utility and use of particular antimicrobials for treatment and control of BRD. Results of this literature review suggest that estimates of resistance in BRD pathogens should consider the context of antimicrobial, pathogen and timing of sample collection, and that continued monitoring of AMR in the BRD pathogens is warranted.

## Figures and Tables

**Table 1:** (**a**) Descriptive analysis of manuscripts reporting antimicrobial susceptibility test data for **florfenicol**; (**b**) Descriptive analysis of manuscripts reporting antimicrobial susceptibility test data for **tildipirosin**; (**c**) Descriptive analysis of manuscripts reporting antimicrobial susceptibility test data for **tulathromycin**.

**florfenicol**						
Pathogen	Total number of reporting manuscripts	References	Total datapoints * for analysis	Minimum isolates tested	Maximum isolates tested	Median isolates tested
*H. somni*	15	[7,8,9,10,11,12,13,14,15,16,17,18,19,20,21]	22	13	459	44
*M. haemolytica*	23	[7,8,9,10,11,12,13,14,15,16,17,18,19,20,21,22,23,24,25,26,27,28,29]	35	5	2989	79
*P. multocida*	17	[7,8,9,10,11,12,13,14,15,16,17,18,19,20,21,22,25]	26	8	1145	87
**tildipirosin**						
Pathogen	Total number of reporting manuscripts	References	Total datapoints * for analysis	Minimum isolates tested	Maximum isolates tested	Median isolates tested
*H. somni*	15	[7,8,9,10,11,12,13,14,15,16,17,18,19,20,21]	22	13	458	44
*M. haemolytica*	24	[7,8,9,10,11,12,13,14,15,16,17,18,19,20,21,22,23,24,25,26,27,28,29,30]	37	5	2989	79
*P. multocida*	17	[7,8,9,10,11,12,13,14,15,16,17,18,19,20,21,22,25]	26	8	1145	87
**tulathromycin**						
Pathogen	Total number of reporting manuscripts	References	Total datapoints * for analysis	Minimum isolates tested	Maximum isolates tested	Median isolates tested
*H. somni*	15	[7,8,9,10,11,12,13,14,15,16,17,18,19,20,21]	22	13	458	44
*M. haemolytica*	24	[7,8,9,10,11,12,13,14,15,16,17,18,19,20,21,22,23,24,25,26,27,28,29,30]	37	5	2989	79
*P. multocida*	17	[7,8,9,10,11,12,13,14,15,16,17,18,19,20,21,22,25]	26	8	1145	87

**florfenicol** * Datapoint—the most granular sub-population for which individual manuscripts reported AST data; datapoints could represent different sample types or time periods within a manuscript. Ex—A manuscript reported the % resistance for florfenicol in each year over a 5-year period, this would represent five datapoints. **tildipirosin** * Datapoint—the most granular sub-population for which individual manuscripts reported AST data; datapoints could represent different sample types or time periods within a manuscript. Ex—A manuscript reported the % resistance for tildipirosin in each year over a 5-year period, this would represent five datapoints. **tulathromycin** * Datapoint—the most granular sub-population for which individual manuscripts reported AST data; datapoints could represent different sample types or time periods within a manuscript. Ex—A manuscript reported the % resistance for tulathromycin in each year over a 5-year period, this would represent five datapoints.

**Table 2:** Adjusted probability (with Standard Error and Upper/Lower 95% Confidence Intervals) of antimicrobial resistance for the given variables for *M. haemolytica*, *P. multocida* and *H. somni* calculated from 24 manuscripts published between 2015–2024.

***M. haemolytica* Models**					
**Antimicrobial**	**Model-estimated probability of resistance**	**SE**	**Lower 95% CI**	**Upper 95% CI**	**††**
Florfenicol	0.0700	0.0230	0.0363	0.1308	^a^
Tildipirosin	0.0629	0.0213	0.0319	0.1200	^a^
Tulathromycin	0.2408	0.0642	0.1375	0.3870	^b^
**Sample timing**					
Arrival	0.0263	0.0107	0.0118	0.0576	^ab^
Arrival and Post-arrival	0.0008	0.0013	0.0000	0.0187	^a^
Diagnostic submissions	0.1890	0.1640	0.0278	0.6549	^bc^
Post-arrival	0.3319	0.0885	0.1852	0.5206	^c^
***P. multocida* Models**					
**Antimicrobial**					
Florfenicol	0.0483	0.0186	0.0225	0.1009	^a^
Tildipirosin	0.2148	0.0689	0.1094	0.3786	^b^
Tulathromycin	0.1049	0.0378	0.0506	0.2051	^c^
**Sample timing**					
Arrival	0.0298	0.0138	0.0118	0.0728	^a^
Arrival and Post-arrival	0.0029	0.0041	0.0002	0.0443	^a^
Diagnostic submissions	0.0929	0.0950	0.0111	0.4828	^ab^
Post-arrival	0.1786	0.0660	0.0826	0.3442	^b^
***H. somni* Models**					
**Antimicrobial**					
Florfenicol	0.0117	0.0048	0.0052	0.0260	^a^
Tildipirosin	0.0519	0.0206	0.0236	0.1104	^b^
Tulathromycin	0.0819	0.0288	0.0404	0.1590	^c^
**Sample timing**					
Arrival	0.0123	0.0062	0.0046	0.0326	^a^
Arrival and Post-arrival	0.0170	0.0190	0.0019	0.1378	^ab^
Diagnostic submissions	0.0904	0.0795	0.0147	0.3982	^ab^
Post-arrival	0.0421	0.0179	0.0180	0.0951	^b^

††—rows with different superscripts are significantly different (*p* ≤ 0.05) within antimicrobial or sample timing category.

## Data Availability

The original contributions presented in this study are included in the article/Supplementary Material. Further inquiries can be directed to the corresponding author.

## Supplementary Materials

The following supporting information can be downloaded at: https://www.mdpi.com/article/10.3390/ani15121789/s1, Table S1: Iterative search strategy after removal of “dairy cattle”, “dairy”, “fungal resistance” and their variant terms and resulting number of articles retrieved; Figure S1: (a) Prevalence of florfenicol resistance reported by individual manuscript and year of isolate collection for *Pasteurella multocida*; (b) Prevalence of tildipirosin resistance reported by individual manuscript and year of isolate collection for *Pasteurella multocida;* (c) Prevalence of tulathromycin resistance reported by individual manuscript and year of isolate collection for *Pasteurella multocida*. Figure S2: (a) Prevalence of florfenicol resistance reported by individual manuscript and year of isolate collection for *Histophilus somni*; (b) Prevalence of tildipirosin resistance reported by individual manuscript and year of isolate collection for *Histophilus somni*; (c) Prevalence of tulathromycin resistance reported by individual manuscript and year of isolate collection for *Histophilus somni*.

## Abbreviations

The following abbreviations are used in this manuscript:

AMR	Antimicrobial Resistance
AST	Antimicrobial Susceptibility Test(ing)
BRD	Bovine Respiratory Disease
