# Supplementary material for: Antimicrobial Resistance in Bovine Respiratory Disease Pathogens: A Systematic Review and Analysis of the Published Literature
Source: Animals (Basel). 2025 Jun 18;15(12):1789. doi: 10.3390/ani15121789 (PMC12189046; doi:10.3390/ani15121789)
Supplement: Supplementary file 1 [file animals-15-01789-s001.zip › Supplemental Table S1.pdf]

Supplemental Table S1. Iterative search strategy after removal of “dairy cattle”, “dairy”, “fungal resistance” and their variant terms and resulting number of articles retrieved.

Search Number	Refinement Parameters & Boolean Logic	Search Terms and Methods	Results
1		cattle	404,883
2		cow	417,670
3		cows	416,178
4		feedlot cattle	3,491
5		beef cattle	25,336
7		heifer	11,769
8		heifers	11,769
9		steer	40,762
10		bull	693,482
11		bos taurus	405,353
12		bovine	488,161
13		feedlot*	4,163
14		beef*	34,832
17		"anti-microbial resistance"	345
21		"antimicrobial resistance"	50,944
23		"bacterial resistance"	10,188
24		"antibiotic resistance"	61,919
26		"resistance genes"	29,576
27		"antibiotic resistance genes"	10,287
28		"antimicrobial resistance genes"	3,442
29		AMR	15,798
31		"anti-microbial susceptibility"	83
32		"antimicrobial susceptibility"	20,526
33		"antibiotic susceptibility"	13,084
34		"anti-biotic susceptibility"	5
35		"minimum inhibitory concentration"	20,616
36		"resistance mechanisms"	16,585
37		"antimicrobial resistance mechanisms"	312
39		mannheimia haemolytica	1,478
40		Pasteurella haemolytica	2,220
41		Pasteurella hemolytica	1,535
42		Pasteurella multocida	4,805
43		Haemophilus somnus	360
44		Histophilus somni	613
45		Hemophilus somnus	362
46		Hemophilus somni	46
47	39OR40OR…46	(((((((mannheimia haemolytica) OR (Pasteurella haemolytica)) OR (Pasteurella hemolytica)) OR (Pasteurella multocida)) OR (Haemophilus somnus)) OR (Histophilus somni)) OR (Hemophilus somnus)) OR (Hemophilus somni)	6,905

48		Tildipirosin	67
49		Zuprevo	67
50		Florfenicol	1,812
51		nuflor	1,812
52		tulathromycin	311
53		draxxin	311
54		increxxa	311
55		macrosyn	311
56	48OR49OR…55	(((((((Tildipirosin) OR (Zuprevo)) OR (Florfenicol)) OR (nuflor)) OR (tulathromycin)) OR (draxxin)) OR (increxxa)) OR (macrosyn)	2,085
58	1OR…14	(((((((((((((cattle) OR (cow)) OR (cows)) OR (feedlot cattle)) OR (beef cattle)) OR (heifer)) OR (heifers)) OR (steer)) OR (bull)) OR (bos taurus)) OR (bovine)) OR (feedlot*)) OR (beef*)	836,367
59	17OR…37	(((((((((((((("anti-microbial resistance") OR ("antimicrobial resistance")) OR ("bacterial resistance")) OR ("antibiotic resistance")) OR ("resistance genes")) OR ("antibiotic resistance genes")) OR ("antimicrobial resistance genes")) OR (AMR)) OR ("anti-microbial susceptibility")) OR ("antimicrobial susceptibility")) OR ("antibiotic susceptibility")) OR ("anti-biotic susceptibility")) OR ("minimum inhibitory concentration")) OR ("resistance mechanisms")) OR ("antimicrobial resistance mechanisms")	177,725
60	58AND59AND47AND56	((((((((((((((((cattle) OR (cow)) OR (cows)) OR (feedlot cattle)) OR (beef cattle)) OR (heifer)) OR (heifers)) OR (steer)) OR (bull)) OR (bos taurus)) OR (bovine)) OR (feedlot*)) OR (beef*)) AND ((((((((((((((("anti-microbial resistance") OR ("antimicrobial resistance")) OR ("bacterial resistance")) OR ("antibiotic resistance")) OR ("resistance genes")) OR ("antibiotic resistance genes")) OR ("antimicrobial resistance genes")) OR (AMR)) OR ("anti-microbial susceptibility")) OR ("antimicrobial susceptibility")) OR ("antibiotic susceptibility")) OR ("anti-biotic susceptibility")) OR ("minimum inhibitory concentration")) OR ("resistance mechanisms")) OR ("antimicrobial resistance mechanisms"))) AND ((((((((mannheimia haemolytica) OR (Pasteurella haemolytica)) OR (Pasteurella hemolytica)) OR (Pasteurella multocida)) OR (Haemophilus somnus)) OR (Histophilus somni)) OR (Hemophilus somnus)) OR (Hemophilus somni))) AND ((((((((Tildipirosin) OR (Zuprevo)) OR (Florfenicol)) OR (nuflor)) OR (tulathromycin)) OR (draxxin)) OR (increxxa)) OR (macrosyn))	69
61	refined for 2015-2024	((((((((((((((((cattle) OR (cow)) OR (cows)) OR (feedlot cattle)) OR (beef cattle)) OR (heifer)) OR (heifers)) OR (steer)) OR (bull)) OR (bos taurus)) OR (bovine)) OR (feedlot*)) OR (beef*)) AND ((((((((((((((("anti-microbial resistance") OR ("antimicrobial resistance")) OR ("bacterial resistance")) OR ("antibiotic resistance")) OR ("resistance genes")) OR ("antibiotic resistance genes")) OR ("antimicrobial resistance genes")) OR (AMR)) OR ("anti-microbial susceptibility")) OR ("antimicrobial susceptibility")) OR ("antibiotic susceptibility")) OR ("anti-biotic susceptibility")) OR ("minimum inhibitory concentration")) OR ("resistance mechanisms")) OR ("antimicrobial resistance mechanisms"))) AND ((((((((mannheimia haemolytica) OR (Pasteurella haemolytica)) OR (Pasteurella hemolytica)) OR (Pasteurella multocida)) OR (Haemophilus somnus)) OR (Histophilus somni)) OR (Hemophilus somnus)) OR (Hemophilus somni))) AND ((((((((Tildipirosin) OR (Zuprevo)) OR (Florfenicol)) OR (nuflor)) OR (tulathromycin)) OR (draxxin)) OR (increxxa)) OR (macrosyn))	48
