# Supplementary material for: Antimicrobial Resistance in Bovine Respiratory Disease Pathogens: A Systematic Review and Analysis of the Published Literature
Source: Animals (Basel). 2025 Jun 18;15(12):1789. doi: 10.3390/ani15121789 (PMC12189046; doi:10.3390/ani15121789)
Supplement: Supplementary file 1 [file animals-15-01789-s001.zip › Supplementary Figures S1abc and S2abc.pdf]

Supplementary Figure S1a. Prevalence of **florfenicol** resistance reported by individual manuscript and year of isolate collection for *Pasteurella multocida*.

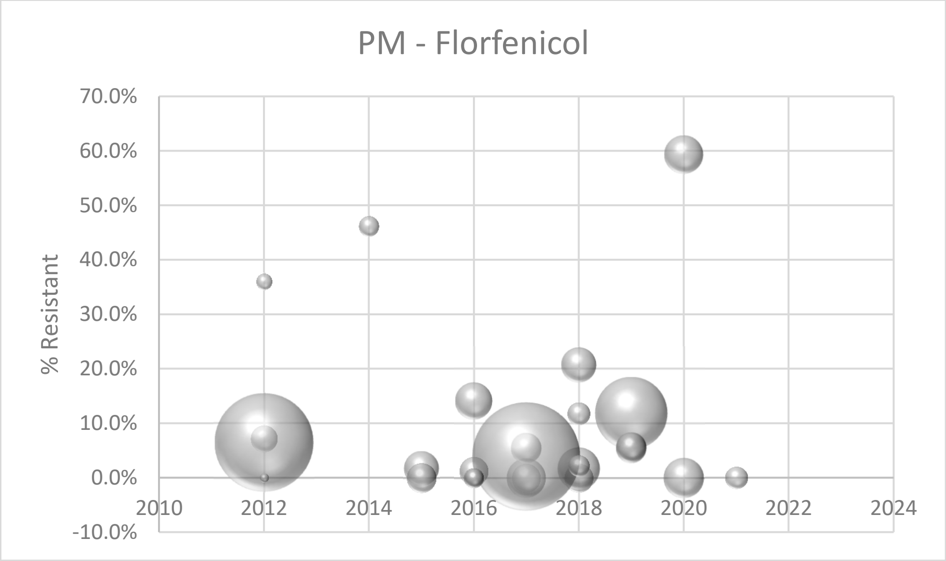


Each point represents the percent of resistant isolates estimated from an individual manuscript. Bubble sizes represent the relative number of isolates in each published study.

Supplementary Figure S1b. Prevalence of **tildipirosin** resistance reported by individual manuscript and year of isolate collection for *Pasteurella multocida*.

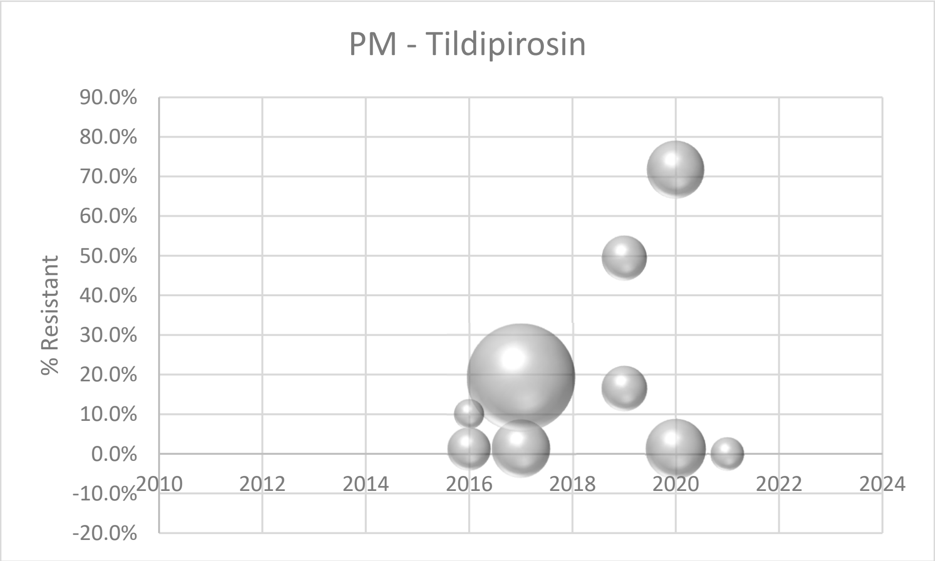


Each point represents the percent of resistant isolates estimated from an individual manuscript. Bubble sizes represent the relative number of isolates in each published study.

Supplementary Figure S1c. Prevalence of **tulathromycin** resistance reported by individual manuscript and year of isolate collection for *Pasteurella multocida*.

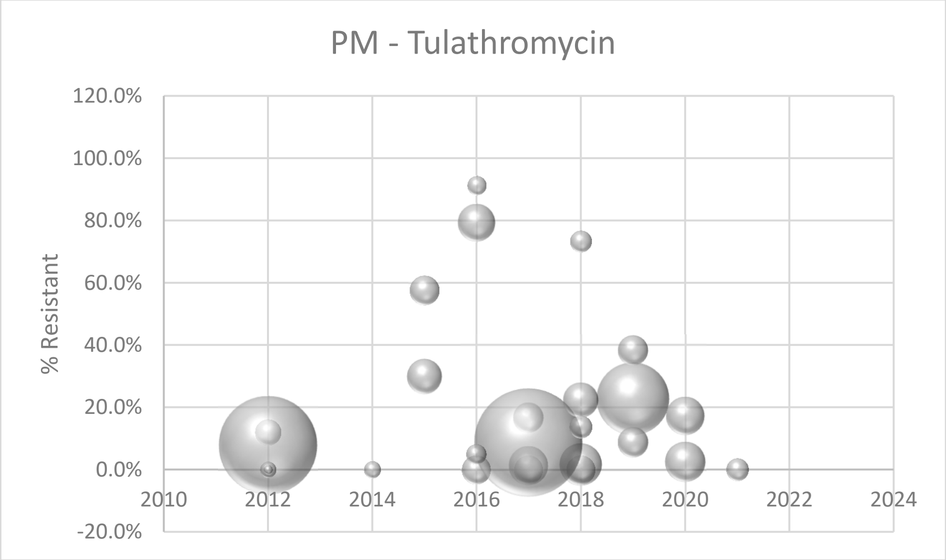


Each point represents the percent of resistant isolates estimated from an individual manuscript. Bubble sizes represent the relative number of isolates in each published study.

Supplementary Figure S2a. Prevalence of **florfenicol** resistance reported by individual manuscript and year of isolate collection for *Histophilus somni*.

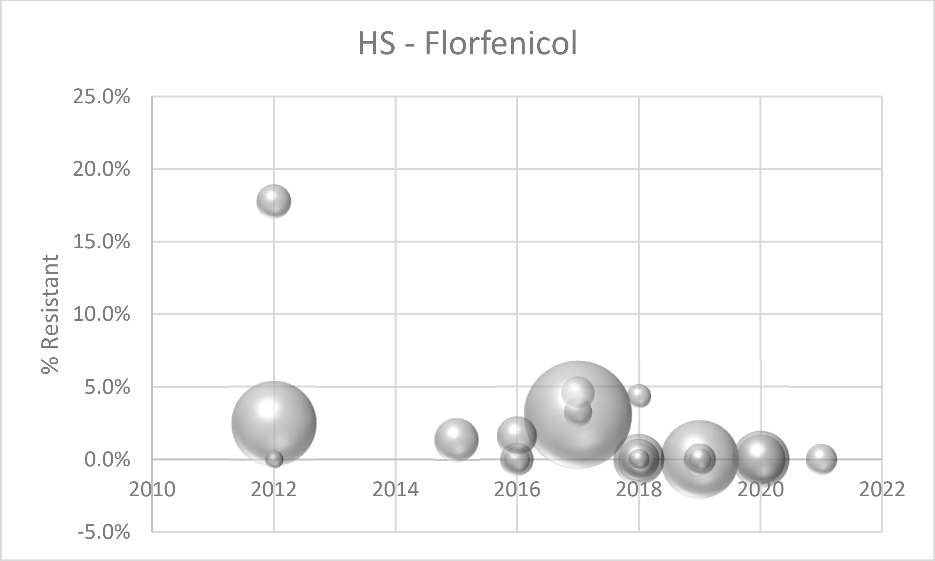


Each point represents the percent of resistant isolates estimated from an individual manuscript. Bubble sizes represent the relative number of isolates in each published study.

Supplementary Figure S2b. Prevalence of **tildipirosin** resistance reported by individual manuscript and year of isolate collection for *Histophilus somni*.

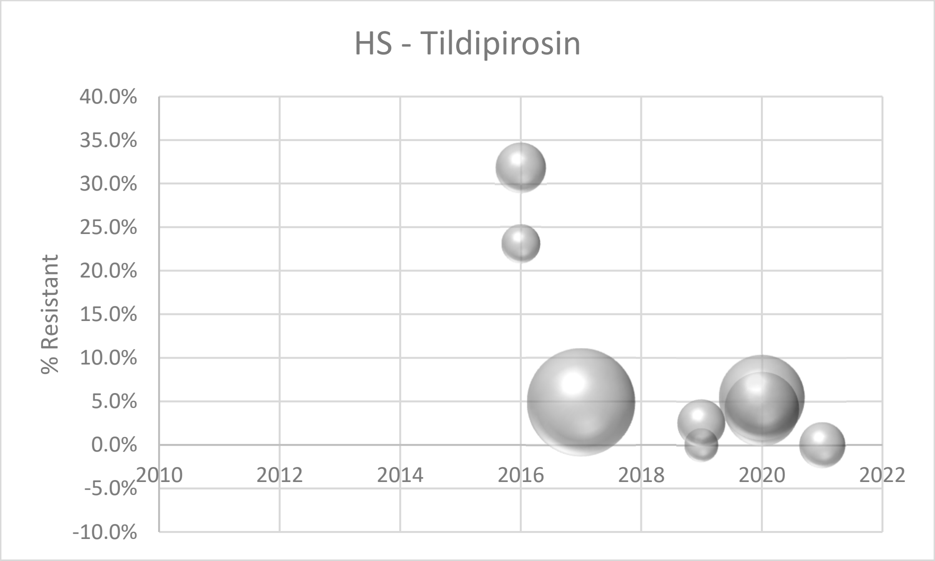


Each point represents the percent of resistant isolates estimated from an individual manuscript. Bubble sizes represent the relative number of isolates in each published study.

Supplementary Figure S2c. Prevalence of **tulathromycin** resistance reported by individual manuscript and year of isolate collection for *Histophilus somni*.

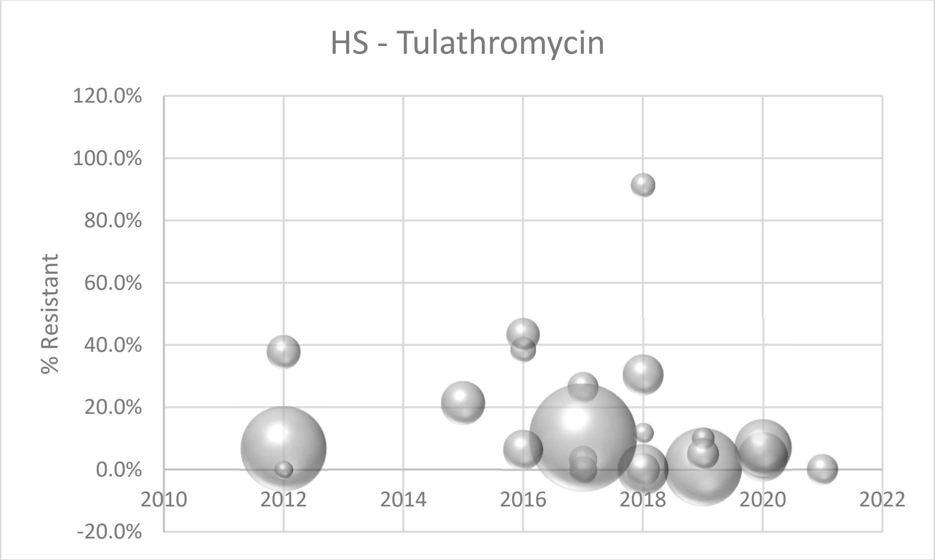


Each point represents the percent of resistant isolates estimated from an individual manuscript. Bubble sizes represent the relative number of isolates in each published study.
